# Investigating Peri-Ictal MRI Abnormalities: A Prospective Neuroimaging Study on Status Epilepticus, Seizure Clusters, and Single Seizures

**DOI:** 10.3390/jcm14082711

**Published:** 2025-04-15

**Authors:** Angelo Pascarella, Lucia Manzo, Oreste Marsico, Emilio Africa, Alessandra Coglitore, Vittoria Cianci, Alessandro Bulgari, Domenico Abelardo, Sara Gasparini, Antonio Armentano, Umberto Aguglia, Giorgi Kuchukhidze, Eugen Trinka, Edoardo Ferlazzo

**Affiliations:** 1 Department of Medical and Surgical Sciences, Magna Græcia University of Catanzaro, 88100 Catanzaro, Italy; o.marsico@neurorc.it (O.M.); a.bulgari@neurorc.it (A.B.); d.abelardo@unicz.it (D.A.); s.gasparini@unicz.it (S.G.); u.aguglia@gmail.com (U.A.); 2 Regional Epilepsy Centre, Great Metropolitan “Bianchi-Melacrino-Morelli Hospital”, 89124 Reggio Calabria, Italy; 3 Department of Neurology, Neurocritical Care and Neurorehabilitation, Christian Doppler University Hospital, European Reference Network EpiCARE, Centre for Cognitive Neuroscience, Paracelsus Medical University of Salzburg, 5020 Salzburg, Austria; g.kuchukhidze@salk.at (G.K.); e.trinka@salk.at (E.T.); 4 Neurology Unit, Great Metropolitan “Bianchi-Melacrino-Morelli Hospital”, 89124 Reggio Calabria, Italy; l.manzo@neurorc.it (L.M.); v.cianci@neurorc.it (V.C.); 5 Neuroradiology Unit, Great Metropolitan “Bianchi-Melacrino-Morelli Hospital”, 89124 Reggio Calabria, Italy; emilioafrica@virgilio.it (E.A.); aless.coglitore@gmail.com (A.C.); antonio.armentano@ospedalerc.it (A.A.)

**Keywords:** convulsive status epilepticus, epilepsy, non-convulsive status epilepticus, peri-ictal DWI abnormalities, peri-ictal FLAIR abnormalities, transient MRI hyperintensity

## Abstract

**Background/Objectives**: Brain magnetic resonance imaging (MRI) often reveals acute peri-ictal abnormalities (PMAs) during or shortly after status epilepticus (SE) but also following single seizures (SiS) or clusters of seizures (CS). However, the incidence, characteristics, and progression remain not clearly known. This study aimed to investigate incidence, clinical correlations, and evolution of PMAs in SE, CS, and SiS patients. **Methods**: This prospective observational study enrolled patients with SE, CS, and SiS who underwent MRI within 120 h of the ictal event. Demographic, clinical, EEG, and MRI data were collected. Patients with PMAs (PMAs+) underwent serial follow-up MRI. Incidence, association with clinical characteristics, and progression of PMAs were analyzed across the three groups. **Results**: Among 76 patients (30 SE, 22 CS, 24 SiS), PMAs were observed in 31 (41%), with a significant difference between groups (*p* = 0.011), as PMAs were less frequent in SiS (17%) compared to SE (57%) and CS (45%) patients. Acute symptomatic SE/seizures were significantly more common in PMAs+ compared to PMAs− in the overall cohort (52% vs. 29%; *p* = 0.045) and in the SiS group (100% vs. 25%; *p* = 0.031). History of epilepsy was less frequent in PMAs+ in the whole cohort (13% vs. 40%; *p* = 0.011) and in SE in particular (12% vs. 46%, *p* = 0.049). No association between PMAs and seizure type, SE duration, etiology, time to MRI, and EEG findings (*p* > 0.005) was found. The temporal cortex and hippocampus were most frequently affected by PMAs. Follow-up MRI performed in 16 patients showed resolution of PMAs in 75% (5/7 SE, 3/6 CS, 3/3 SiS) within a median time of 24 days (IQR: 8–39). **Conclusions**: PMAs were more common in SE and CS than in SiS. Acute underlying pathology was frequently associated with PMAs. While duration of ictal activity is an important factor, it was not the sole determinant. Most PMAs resolved, particularly in SiS. Further studies are needed to clarify the pathophysiological mechanism and clinical implications of PMAs.

## 1. Introduction

In recent years, the increasing use of brain magnetic resonance imaging (MRI) for seizure evaluation has led to the identification of acute brain changes occurring during the ictal or early postictal phase, particularly in the context of status epilepticus (SE) [1,2,3,4]. Peri-ictal MRI abnormalities (PMAs) typically present as restricted diffusion on diffusion-weighted imaging (DWI) with corresponding low apparent diffusion coefficient (ADC) values or as high signal intensity on Fluid Attenuated Inversion Recovery (FLAIR) and T2-weighted sequences [5,6,7,8]. These imaging patterns suggest a spectrum of cytotoxic and vasogenic edema influenced by SE duration and MRI timing [9,10]. Ictal hyperperfusion is also commonly detected on MR perfusion sequences, such as perfusion-weighted imaging (PWI) and arterial spin labeling (ASL) [4,11]. PMAs often localize in cortical regions involved during the seizures but can also affect remote, network-related areas, including the ipsilateral pulvinar nucleus of the thalamus, contralateral cerebellum, basal ganglia, claustrum, and ipsilateral hippocampus [8,12,13]. While these signal changes are frequently transient, resolving on follow-up MRI [3,4,5,14,15,16], they can result sometimes in permanent structural damage, such as cortical laminar necrosis, hippocampal sclerosis, and focal brain atrophy [6,8,16,17,18,19]. Although PMAs are most commonly observed following SE, they have also been documented after a single seizure (SiS) or a cluster of seizures (CS) [20,21,22,23,24,25]. Reported incidence rates of PMAs in SE vary widely, from 12% to 60% [6,8,14,23,26,27,28,29,30], while after a single seizure, the rates are much lower, ranging between 0.007% and 3% [24,31]. However, the precise incidence of PMAs, their characteristics, progression, and underlying mechanisms remain incompletely understood. Indeed, much of the existing knowledge stems from case reports and small retrospective studies, which often have methodological limitations, such as heterogeneous patient populations, variability in MRI timing, and lack of standardized imaging protocols. Prospective data are scarce, and no large-scale studies have comprehensively addressed these aspects [4,23,32,33]. Data regarding PMAs following single or cluster seizures are even scarcer [22,23,24].

This prospective, longitudinal study aims to assess the incidence of PMAs in patients experiencing SiS, CS, and SE. This study also investigates the relationship between PMA topographical characteristics, association with seizures or SE clinical features, and PMA evolution across the patient groups. We hypothesized that PMAs could also be detected in a substantial proportion of CS and SiS patients, though to a lesser extent in the latter, if MRI is performed within an appropriate timeframe. Additionally, we explored whether PMA and its evolution were influenced by factors such as the etiology and duration of the ictal event, as well as the timing of the MRI.

## 2. Materials and Methods

### 2.1. Study Design and Participants

In this prospective, observational, monocentric study, we enrolled consecutive adult patients (age > 16 years) who presented to the Emergency Department and to the Regional Epilepsy Centre of the Great Metropolitan “Bianchi-Melacrino-Morelli Hospital” of Reggio Calabria, Italy, between February 2020 and August 2023, due to the occurrence of seizures or SE and who underwent a brain MRI either during SE or within 120 h after the ictal event (SE, CS, or SiS).

Participants were selected based on the following inclusion criteria: (a) occurrence of a SiS, CS (defined as two or more seizures within the last 24 h with full recovery in between, established through clinical assessment—i.e., return to baseline conditions, no evident critical manifestations—and EEG—absence of ictal activity), or SE (either convulsive, CSE, or non-convulsive, NCSE) diagnosed through clear clinical history and/or well-documented EEG, according to International League Against Epilepsy (ILAE) definitions [34]; and (b) MRI scan performed during SE or within the first 120 h after the onset of ictal events. Exclusion criteria included (a) evidence of central nervous system infection (confirmed by cerebrospinal fluid abnormalities), hypoxic-ischemic encephalopathy, or underlying structural acute brain that could potentially compromise the accurate interpretation of the MRI imaging; and (b) presence of contraindications to MRI (such as pacemakers, MR-incompatible prosthetic heart valves, or metallic implants) or critical illness (e.g., severe respiratory or cardiovascular instability) not allowing one to perform an MRI.

### 2.2. Procedure

#### 2.2.1. Patients’ Clinical Data

At baseline, we collected demographic and clinical data, including previous epilepsy diagnoses, comorbidities, semiology, duration, and etiology of seizures/SE. Seizures and SE were classified according to the ILAE classification system [34,35]. During hospitalization, all patients received treatment following standard guidelines. Additionally, all patients underwent video-EEG examination (Micromed, 21 channels, International 10–20 System; Micromed, Mogliano Veneto, Italy) either before or shortly after MRI to confirm SE diagnosis and exclude NCSE or ictal-interictal continuum in patients with CS and SiS. EEG were evaluated by three board-certified neurologists (SG, UA, and EF) and classified according to the current terminology of the American Clinical Neurophysiology Society Consensus and Salzburg’s Consensus EEG Criteria.

#### 2.2.2. Brain MRI

All participants underwent brain MRI performed on a 1.5 Tesla System (General Electric, Fairfield, CT, USA). The MRI protocol included axial DWI sequences acquired with a 5 mm slice thickness, axial T2-weighted Turbo Spin-Echo (TSE) with slice thickness of 5 mm, axial T1 Spin-Echo (SE) sequence with slice thickness of 5 mm, 3D fluid-attenuated inversion recovery (FLAIR) acquired with isotropic millimetric voxel resolution, and Inversion recovery (IR)—a conventional spin echo (SE) sequence preceded by a 180° inverting pulse—with slice thickness of 3 mm. ADC maps were obtained from DWI. All DICOM data were encrypted and sent telemetrically to a central server for storing and interpretation. MRI scans were independently evaluated by two experienced neuroradiologists (EA and AC) who were blinded to the clinical details (i.e., SE, CS, or SiS) as well as the timing (baseline or follow-up). In the case of disagreements, any divergences were resolved through consultation with a third reviewer (AA). The neuroradiologists examined DWI, ADC, and FLAIR scans for identifying the presence and location of signal or morphological brain abnormalities attributable to PMA, namely acute, transient brain changes occurring during the ictal or early postictal phase reflecting seizure-induced alterations [2,3,4]. Identified MRI abnormalities were attributed to PMA if they (a) involved a cortical region or an adjacent subcortical area not respecting vascular territories; (b) involved the hippocampal area, pulvinar thalami, or the claustrum/peri-insular regions, a contralateral cerebellar area (considered to be crossed cerebellar diaschisis); (c) were hyperintense in DWI (DWI b-value of 1000) and hypointense in the ADC map, and/or hyperintense in FLAIR sequences. Patients with underlying conditions that might present with locally increased signal in FLAIR and DWI (such as neoplasia, encephalitis, infections, or strokes), where the abnormalities were in the same region as the primary lesion and could not be clearly attributable to the ictal event (SE, CS or SiS), were excluded. Follow-up MRI scans were performed in patients showing PMAs at baseline and repeated until the PMAs disappeared, adhering as much as possible to the following time schedule: 1 weeks ± 2 days, 2 weeks ± 2 days, 4 weeks ± 5 days, and 12 weeks ± 10 days after the last seizure or SE termination. However, follow-up MRI data that did not adhere to the scheduled timeframes were also included in the analysis. According to the study protocol, data from patients presenting additional SE or a cluster of seizures during the follow-up period, as well as a single seizure during the week before the scheduled MRI, were to be excluded from the analysis; however, no patients were excluded due to these reasons. The study protocol, including the MRI timing, was designed based on the data available in the literature.

### 2.3. Standard Protocol Approval

The study received approval from the local Ethical Committee (Comitato Etico Sezione Area Centro Regione Calabria, protocol approval number: 115/19, date: 18 April 2019) and was conducted in accordance with the Declaration of Helsinki. Informed written consent was obtained from each patient or their parents or a legal representative.

### 2.4. Statistical Analysis

According to seizure presentation, the patients were grouped as follows: (1) SE; (2) CS; (3) SiS. According to MRI findings, they were classified into two groups: (1) with PMAs (PMAs+); (2) without PMAs (PMAs−). Descriptive data were presented as numbers and percentages for categorical variables, and as mean ± standard deviation (SD) or median with interquartile range (IQR) for continuous variables, as appropriate. The normality of all variables considered was assessed using the Kolmogorov–Smirnov test. Patients with PMAs were compared to those without PMAs, and additional comparisons were performed among the three seizure groups. Pair-wise analyses were conducted to compare SE vs. CS, SE vs. SiS, and CS vs. SiS; the significance level was corrected using the Bonferroni method to control for multiple testing. Differences between groups were assessed with a *t*-test, ANOVA, Kruskal–Wallis test, Mann–Whitney U test, chi-squared test, or Wilcoxon test, as appropriate. Statistical significance was set at *p* < 0.05. Statistical analyses were performed using SPSS 29.0.2.0 (IBM StataCorp LP, College Station, TX, USA).

## 3. Results

### 3.1. Study Population

A total of 91 consecutive patients with SiS, CS, or SE who underwent an MRI within 120 h from seizure onset were enrolled during the recruitment period. Of them, fifteen patients were excluded from the study because of MRI changes not clearly distinguishable from a concomitant or pre-existing primary brain lesion. Thus, the final sample consisted of 76 patients (39 females, 51%; median age: 71 years, IQR: 53–81), of whom 30 had SE (15 females, 50%; median age: 77 years, IQR: 66–84), 22 had CS (16 females, 73%; median age: 73 years, IQR: 59–82) and 24 had a SiS (8 females, 33%; median age: 58 years, IQR: 44–70; Figure 1; Table 1).

Patients in the SiS group were younger (*p* = 0.001, Table 2) as compared to both SE (*p* = 0.001 in pair-wise analysis) and CS (*p* = 0.021 in pair-wise analysis) patients; moreover, the SiS group had a more prevalent male population as compared to patients with CS (pair-wise analysis: *p* = 0.010). No statistically significant difference in median time to MRI (24 h—IQR: 7–36.75—for SE patients, 7 h—IQR: 5–36.5—for CS patients, 7 h—IQR: 5–26.75—for those with SiS; *p* = 0.102) was found. The most frequent types of SE were focal motor (13/30, 43%) and convulsive (11/30, 37%), with a mean duration of 55.6 ± 65 h (range: 1–240 h; Table 2). Twenty-two (73%) of the patients with a SE had no prior history of epilepsy (“new onset SE”, NOSE). In terms of etiology, the most frequent cause of SE was acute symptomatic origin (12/30, 40%) patients.

In both the CS and SiS groups, the majority presented with generalized seizures (10/22, 45%, and 11/24, 46%, respectively). Similarly, structural etiology was the most common in both groups, occurring in 14 (64%) of the CS patients and 16 (67%) of the SiS patients (see Table 2 for details), with acute symptomatic origin in 6 (27%) and 11 (46%) patients, respectively. Only five (23%) of the CS patients had a previous history of epilepsy, while epilepsy diagnosis was reported in nine (35%) of the SiS patients. Six patients (four SE, one CS, and one SiS) died during the follow-up period. Two of the four SE patients died during refractory SE: one was an 80-year-old male with no prior history of epilepsy who died 80 h after FMSE onset due to a symptomatic acute vascular lesion; the second was an 85-year-old female, also without a history of epilepsy, who died 240 h after NCSE onset, attributed to a remote structural etiology (ischemic stroke). The remaining two SE patients died after FMSE resolution and hospital discharge due to unknown causes; neither had a history of epilepsy. The patient with CS experienced an acute symptomatic seizure secondary to an infectious etiology and died due to infectious-related complications. The SiS patient had an acute symptomatic seizure secondary to an ischemic stroke and died from stroke-related complications.

### 3.2. Baseline MRI Findings

We found PMAs in 31 out of 76 (41%) patients at baseline. See Supplementary Figure S1 for MRI images of three representative cases of PMA, one for each study group, and Supplementary Figure S2 for a distinctive and unusual PMA finding in a SiS patient. Occurrence of PMA was significantly less frequent in SiS patients (4/24, 17%) compared to SE and CS patients (17/30, 57% and 10/22, 45%; *p* = 0.011; Table 3). Pairwise analysis revealed a statistically significant difference between SiS and SE patients (*p* = 0.003), but not between SE and CS (*p* = 0.424) or CS and SiS (*p* = 0.54) patients. There was no significant difference in sex distribution between PMAs+ and PMAs− subjects (*p* > 0.05), while the median age was significantly higher in PMAs+ subjects compared to PMAs− (*p* = 0.036). A history of epilepsy was significantly less frequent among PMAs+ patients (4/31, 13%) compared to PMAs− (18/45, 40%; χ^2^ = 6.552, *p* = 0.011). Acute symptomatic seizures were more frequent in PMAs+ individuals as opposed to PMAs− (16/31, 52% vs. 13/45, 29; χ^2^ = 4.017, *p* = 0.045; Table 3). No statistically significant difference in time to MRI (median time: 7 h—IQR: 7–46—vs. 14 h—IQR: 5–32-; *p* = 0.940) was found.

#### 3.2.1. Subgroups Analysis

In patients with SE, no statistical difference was found when comparing PMAs+ and PMAs− in terms of sex, age, SE type and etiology, acute symptomatic etiology, SE duration, and time to MRI (all *p* > 0.05; Table 4). Patients with PMAs less frequently had a history of epilepsy (χ^2^ = 4.455, *p* = 0.049). We observed restricted diffusion on DWI in 15/30 patients (50%), with low signal in the corresponding ADC maps in 11 subjects (37%); FLAIR hyperintensities occurred in 16 (53%) patients; 10 (33%) patients showed abnormalities in both MRI sequences (DWI and FLAIR).

In CS subgroup, PMAs+ and PMAs− patients did not differ in terms of age, sex, etiology, history of epilepsy, acute symptomatic etiology and time to MRI (Table 4). Restricted diffusion was found in 8/22 (36%) patients, with corresponding ADC low signal in 6 (27%) and FLAIR abnormalities in 10 (45%) patients.

No difference was evident between PMAs+ and PMAs− patients in the SiS subgroup for all the considered variables (age, sex, etiology, history of epilepsy, type of seizure), except for the finding of acute symptomatic etiology in all four PMAs+ individuals (χ^2^ = 5.673, *p* = 0.031; Table 4). We observed FLAIR abnormalities in all four PMAs+ patients, while only three of them (12%) showed diffusion restriction with decreased signal in ADC.

#### 3.2.2. Location of PMA

**Figure 2:**
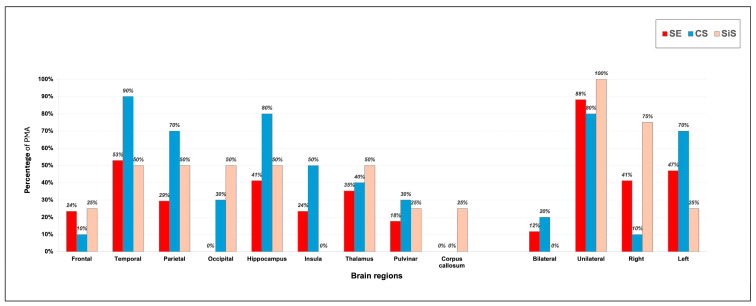
**Anatomical distribution of peri-ictal MRI abnormalities.** The temporal lobe and hippocampus were the most frequently affected regions in SE, CS, and SiS. Thalamic involvement was also frequently observed. In all three groups, the lesions were predominantly unilateral.

The temporal lobe cortex and hippocampus were the regions most frequently affected by PMA in both the SE and CS groups (9/17, 53% and 7/17, 41% in SE patients, and 9/10, 90% and 8/10, 80% of CS patients with PMAs, respectively; Figure 2), whereas no specific location patterns of PMAs were observed in the SiS group. The thalamus was involved in 6/17 (35%) SE patients, 4/10 (40%) CS patients, and 2/4 (50%) SiS patients, with pulvinar involvement in 3 (18%), 3 (30%) and 1 (25%) case, respectively. The lesions were mostly unilateral in all three groups (5/17, 88% in SE patients, 8/10, 80% in CS patients, and all 4 (100%) SiS patients), without any significative statistical differences (χ^2^ = 1.010, *p* = 0.604).

### 3.3. MRI Findings at Follow-Up

**Figure 3:**
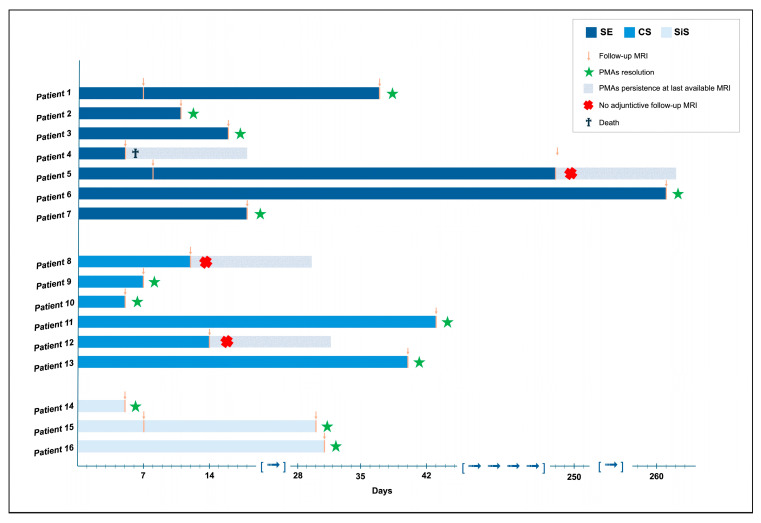
**Timeline of PMA relapse in patients performing follow-up MRI in the SE, CS, or SiS groups.** The figure illustrates the follow-up MRI outcomes for the 16 patients who underwent the procedure (7 SE, 6 CS, 3 SS). Resolution of PMAs was observed in patients 2, 3, 6, 7, 9, 10, 11, 12, 14, and 16 after the first follow-up MRI, while the remaining patients continued to exhibit PMAs. The disappearance of PMAs was demonstrated in patients 1 and 15 after additional MRI scans. Further follow-up was not conducted for four patients due to unavailability (patients 5, 8, and 12) or death (patient 4).

At least one follow-up MRI was performed in 16/31 (52%) patients with PMA; in detail, it was done in 7/17 individuals with SE, 6/10 with CS, and 3/4 with SiS. The reason for not performing follow-up MRIs was death in four patients (three with SE and one with a CS) and refusal to perform the MRI after hospital discharge for eleven patients (seven SE, three CS and one SiS). First follow-up MRI median time from event onset was 11 days (IQR: 7–38; range: 5–261 days). Seven patients underwent the first follow-up within seven days from ictal event onset, four others within two weeks, and five only later. Among the MRIs performed within one week, PMAs disappeared in three patients (56%; SE: 0/3, CS: 2/2, SiS: 1/2) but remained visible in four patients (44%, SE:3/3, CS: 0/2, SiS:1/2; see Figure 3). In three of them (two SE and one SiS; one SE died), subsequent MRI were performed (two within one month, one after eight month), in which PMA disappeared in two of them within one month (one SE, one SiS) (Figure 3). No further follow-up MRI was available for the patient showing PMA persistence. Considering patients with the first follow-up MRI performed within two weeks, three patients (60%; SE: 3/3, CS: 0/2) had normal MRI; and two patients with CS showed persisting PMAs; however, subsequent follow-up MRI was not performed. When the first follow-up MRI was performed after 1 month, images demonstrated PMA resolution in 4/4 patients (one SE, two CS, one SiS).

Hence, the resolution of PMAs was observed in 12/16 (75%) patients (5/7 SE, 4/6 CS, 3/3 SiS). For these four subjects in whom we failed to demonstrate PMA resolution, the last available follow-up MRI was performed 40 and 248 days after SE onset, respectively, and 12 and 14 days after CS onset.

The median time of PMA resolution was 24 days (IQR: 8–39.25, range: 5–261). In detail, the median time of PMA disappearance was 18 days (IQR: 13.5–149, range: 11–261) in the SE group, 23.5 days (IQR: 5.5–42.25, range: 5–43) in the CS group, and 30 days (IQR: 5–n.a., range: 5–31 days) in the SiS group, without statistical difference between the three groups (*p* = 0.587).

## 4. Discussion

In this prospective study, we investigated the occurrence of PMA in patients with SE, CS, and SiS. Our findings provide insights into the incidence, features, and progression of PMAs, highlighting differences between these groups and contributing to a better understanding of the existing gaps in the literature. While previous research was predominantly focused on SE, data regarding CS and SiS remain limited. Moreover, most existing studies have been retrospective, with only a few prospective studies conducted [4,23,32,33,36]. These studies often enrolled SE, CS, and SiS patients into combined heterogeneous cohorts, analyzing them collectively rather than as distinct groups [22,23,24,31]. See Supplementary Tables S1 and S2 for a comprehensive review of the available evidence from prospective studies on PMA in SE, as well as studies involving CS and SiS. To the best of our knowledge, this is the first study to simultaneously enroll and compare patients from all three groups using uniform consistent inclusion criteria and a standardized protocol, effectively addressing a critical limitation in previous research. The lack of uniform inclusion criteria and standardized methodologies across prior studies hinder direct and meaningful comparisons between these distinct groups. By employing a unique, rigorous, and consistent approach, our study uniquely bridges these gaps, offering a more comprehensive understanding of PMAs across the SE, CS, and SiS populations.

Our findings indicate that PMAs are more frequent in SE patients, with over half of this group showing abnormalities. PMAs were also prevalent in the CS group, being present more than one-third of patients, while a much smaller percentage of SiS patients exhibited abnormalities. This gradient highlights the pivotal role of seizure severity and duration in driving acute brain changes.

Findings from our study demonstrated a relatively higher rate of PMA compared to data from retrospective studies on SE patients, where highly variable reported rates range between 11.6% and 63% [8,14,26,27,28,29,30]. The observed inconsistencies can likely be attributed to differences in study designs and methodologies, particularly the lack of standardized timing between SE onset and MRI in many retrospective studies.

Notably, data from our SE cohort better align with previous prospective studies, which have reported PMA rates ranging from 45% to 65% [8,31,32,33,36]. For instance, Bosque Varela et al. identified PMAs in 45% of 206 prospectively recruited SE patients who underwent an MRI [8], while Sarria-Estrada et al. found a similar prevalence of 51.7% in a cohort of 60 patients with focal SE [36]. Differences in methodology between these studies and ours may partially explain the slight variations in PMA frequency, as Bosque Varela’s study included patients imaged at a median of 16 h post-SE onset (IQR 0.25–373 h; 84% of patients underwent an MRI within 48 h after SE onset), while Sarria-Estrada’s analysis included only focal SE cases [8,36]. More frequent occurrence has been observed in three Indian studies, which reported PMA rates ranging from 61.9% to 65.2% [31,32,33]. These elevated rates may reflect differences in study populations (including mainly patients with convulsive SE) and an SE definition (“continuous convulsive activity lasting for more than 10 min” [32]) that does not align with the current ILAE criteria [34].

In the CS group, we observed a PMA incidence of approximately 43%, which is notably lower than the 83.3% reported by Jabeen et al. in their small prospective cohort of six patients [23]. This discrepancy can likely be attributed to the methodological differences, such as the timing of MRI (within 24 h in their study compared to 120 h in ours) and the higher seizure burden in their cohort (10–15 seizures within 48 h). Additional evidence for PMA occurrence in CS patients comes from case reports [37] and retrospective studies on mixed cohorts that also included SE and SiS patients [22,24,31] and so provided limited specific data on the CS population due to the heterogeneity of their samples.

PMAs have also been documented following single seizures [20,21,22,24,31]. To our knowledge, our study is the first to prospectively investigate the occurrence of PMAs in SiS patients. Recognizing PMAs after a seizure is crucial, as they can mimic underlying causes such as infections, inflammation, trauma, or neoplastic etiologies [22]. In our cohort, the incidence of PMAs was relatively low (16%) compared to SE and CS groups but remains noteworthy when compared to the 3% reported in a prior retrospective study involving 416 patients who underwent MRI within 24 h after a single seizure [24]. However, that study only analyzed the DWI sequence and narrowly classified abnormalities as seizure-related (limited to cortical regions, adjacent subcortical areas, or restricted to the hippocampus or pulvinar of thalami), potentially underestimating the prevalence of PMA [24].

Regarding the anatomical distribution of PMAs, most lesions were unilateral, with no correlation identified between lesion location and seizure type (e.g., focal or generalized SE or seizures), aligning with previous studies [28]. The temporal lobe cortex and hippocampus were the most frequently affected regions in patients with SE and CS, as well as in SiS patients, consistent with the literature [4,8,25,28,29,38]. This observation reflects the known vulnerability of mesio-temporal structures to excitotoxic damage [28,39] and their central role in epilepsy, even when they are not the primary epileptogenic site [40]. Similarly, the thalamus, particularly the pulvinar, was frequently involved, likely due to its extensive cortico-thalamic and temporo-parietal connections, which facilitate seizure propagation [4,28,41]. The basal ganglia were also implicated, potentially reflecting their influence on seizure dynamics and involvement in propagating epileptic discharges [42,43]. Additionally, lesions were observed in remote, seemingly unrelated regions, possibly caused by widespread neuronal activity or propagation pathways, akin to the ‘kindling’ model of epilepsy [44].

Interestingly, we did not find a significant correlation between SE duration and PMA occurrence. This finding challenges the widely held assumption that PMAs are strictly duration dependent. While previous studies and experimental research have suggested that SE duration is a critical factor driving MRI-detected changes [23,29,30,36,38], our results indicate that the duration of ictal activity is relevant but not the sole determinant. Other variables likely contribute to the neuronal changes induced by sustained seizure activity and subsequent development of PMAs, though these factors remain largely unknown. This hypothesis is further supported by the observation that PMAs are also detectable in CS and SiS, which are characterized by shorter, lower intensity, and non-continuous clinical manifestations. Among the variables analyzed, no specific seizure type, etiology, or metabolic disorder was found to be significantly associated with PMA development. However, acute symptomatic etiology and the absence of a prior epilepsy history were more strongly linked to PMAs across the sample. This trend was observed consistently in all three subgroups, although not always reaching statistical significance, suggesting that the presence of an underlying pathology may play a more pivotal role. Our findings align with previous studies reporting DWI changes in symptomatic epilepsies or SE cases [15,41,45,46]. This raises the possibility that PMAs, in some cases, may reflect pre-existing vulnerabilities related to the etiology of SE, CS, or SiS, rendering the brain more susceptible to cytotoxic or vasogenic damage [47,48], rather than being solely a direct consequence of seizures or their duration. Likewise, symptomatic etiologies may also result in more pronounced MRI alterations compared to idiopathic conditions, which are presumed to benefit from intrinsic protective mechanisms [45].

Consistent with the literature, our study confirms that PMAs are predominantly transient, resolving in most cases within weeks to months. Follow-up imaging revealed complete resolution in 75% of patients, with a median resolution time of 24 days. Among SiS patients, all abnormalities resolved, whereas resolution occurred in approximately 70% of SE and CS patients with at least one follow-up MRI. However, for CS patients, only short-term follow-up imaging (<2 weeks) was available for the two cases without resolution after first evaluation, leaving uncertainty about whether longer observation would have demonstrated reversal. Conversely, in SE patients with persistent PMAs, the last available MRI was conducted over 40 days after seizure onset, making definitive conclusions and intergroup comparisons difficult. The timeline for PMA resolution varies widely across studies, likely due to differences in study populations and imaging protocols; hence, the precise point at which seizure-induced MRI abnormalities become irreversible remains an open question. In our cohort, 55.6% resolved within one week, and 75% resolved within 14 days. Unfortunately, follow-up MRIs were not performed in all patients within 7 or 14 days, and serial imaging at scheduled times was available in only a minority of cases, particularly in the SE and CS groups. This limited our ability to draw precise conclusions about the exact timeline for normalization. With regard to the specific three groups, partial or complete resolution within days to weeks has been widely documented in SE patients [23,41,45,49], consistent with our findings. Data for CS patients are limited, with resolution reported after three months in a case report [39] and within 45 days in a mixed cohort of CS and SiS patients [22]. Similar to previous studies [25], in our study, follow-up MRIs showed resolution within 5–31 days after a single seizure, although longer resolution times, up to 150 days, have been reported [21].

The present study has some limitations. The relatively small sample size limits the generalizability of our findings, particularly in the subgroup analysis. Additionally, the absence of ASL sequences may have led to an underestimation of detectable changes. Variability in the timing of follow-up MRIs among patients and missing follow-up MRI data likely influenced the observed rates and timing of PMA resolution, limiting the ability to draw definitive conclusions about this aspect. Efforts to adhere to the proposed timing schedule as closely as possible were made; however, this was not always achievable due to logistical constraints, including MRI availability, or patient-related factors, such as their inability or unwillingness to return for follow-up imaging after hospital discharge. Finally, every effort was made to ensure the internal validity of the study, including careful study design, appropriate sampling, controlling for confounding factors, ensuring reliable measurements, and minimizing biases; the single-center setting may affect the external validity. Future and larger multicenter studies with standardized imaging protocols are needed to better clarify unresolved issues.

## 5. Conclusions

This study demonstrates that PMAs are common in SE and CS and, although relatively rare, occur more frequently than expected in SiS. Abnormalities are largely reversible and appear not to be solely dependent on seizure duration, highlighting the potential contribution of underlying pathologies or other still unknown factors as determinants for brain tissue vulnerabilities. These findings underscore the value of MRI in detecting seizure-related brain changes and could provide valuable insights into the mechanisms underlying PMAs. Future research, particularly large-scale studies employing standardized imaging protocols, focusing on the clinical significance of PMAs, their association with long-term outcomes, and their potential role in guiding patient management are needed. These advancements could lead to more personalized care strategies for patients with SE, CS, and SiS.

## Figures and Tables

**Figure 1:**
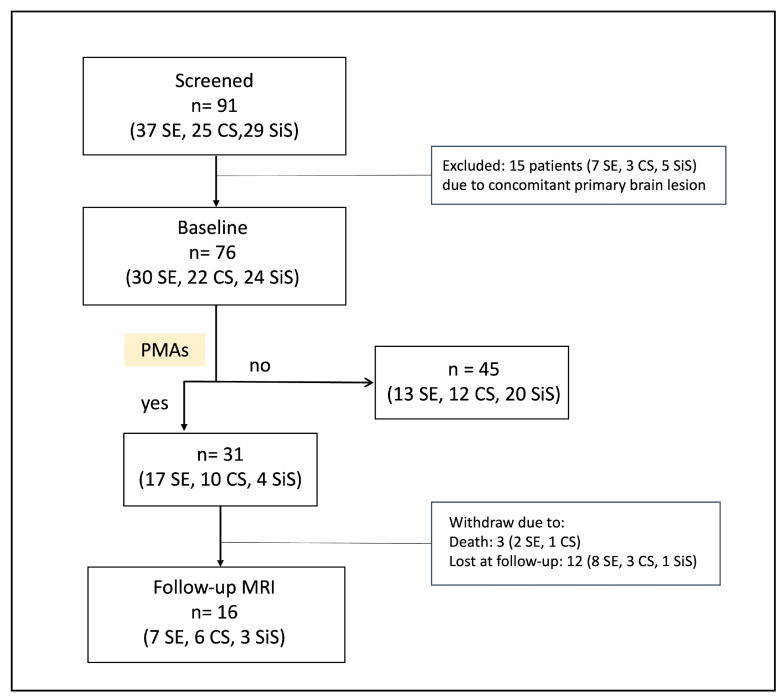
**Study flowchart.** Ultimately, 15 out of the 91 initially enrolled patients were excluded due to overlap of DWI/FLAIR abnormalities with a concomitant or pre-existing primary brain lesion not related to ictal activity. Follow-up MRI was unavailable for 15 subjects.

**Table 1:** Demographic, clinical, and neurophysiological data of the whole study population.

Characteristics	Patients (*n* = 76)
Sex: female/male, *n* (%)	39/37 (51/49)
Age: median (IQR), years	71 (53–81)
History of epilepsy, *n* (%)	22 (29)
Diagnosis: SE, CS, SiS, *n* (%)	30/22/24 (39/29/32)
Acute symptomatic etiology, *n* (%)	29 (38)
Time to MRI: median (IQR), hours	10 (7–33)

CS: cluster of seizures; SE: Status Epilepticus; SiS: single seizure.

**Table 2:** Demographic, clinical, and neurophysiological data of the three groups.

Patients’ Characteristics	SE (*n* = 30)	CS (*n* = 22)	SiS (*n* = 24)	*p*
Sex: female/male, *n* (%)	15/15 (50/50)	16/6 (73/27)	8/16 (33/67)	**0.029 ^#^**
Age: median (IQR), years	77 (66–84)	73 (59–82)	58 (44–70)	**0.001 ^§^**
History of epilepsy, *n* (%)	8 (27)	5 (23)	9 (37)	0.511
Acute symptomatic seizures, *n* (%)	12 (40)	6 (27)	11 (46)	0.417
Status Epilepticus type, *n* (%)				
Convulsive	11 (37)	-	-	
Myoclonic	1 (3)	-	-	
Focal motor	13 (43)	-	-	
Non convulsive	5 (17)	-	-	
Seizure type, *n* (%)				
Generalized	-	10 (45)	11 (46)	
Focal	-	4 (18)	8 (33)	
Focal to bilateral	-	8 (36)	4 (17)	
Unknown	-	0	1 (4)	
Status Epilepticus etiology, *n* (%)				
Acute	12 (40)	-	-	
Remote	11 (37)	-	-	
Progressive	2 (7)	-	-	
In defined electroclinical syndromes	1 (3)	-	-	
Unknown	4 (13)	-	-	
Seizure etiology, *n* (%)				
Structural	-	14 (64)	16 (67)	
Vascular	-	12 (54)	12 (50)	
Tumoral	-	1 (4)	2 (8)	
Malformative	-	0	1 (4)	
Degenerative	-	1 (4)	1 (4)	
Genetic	-	0	1 (4)	
Metabolic	-	1 (4)	0	
Infectious	-	3 (14)	0	
Autoimmune	-	1 (4)	0	
Unknown	-	3 (14)	7 (32)	
SE duration: median (IQR), hours	30 (4–74)	-	-	
Time to MRI: median (IQR), hours	24 (7–37)	7 (5–36)	7 (5–27)	0.102

In bold: significantly statistical differences. ^#^ Pairwise analysis: CS vs. SiS: *p* = 0.010; ^§^ Pairwise analysis: SE vs. SiS: *p* = 0.001; CS vs. SiS: *p* = 0.021.

**Table 3:** MRI findings at baseline in the whole populations.

Total Sample (*n* = 76)		PMA+	PMA−	*p*
Patients, *n.* (%)		31 (41)	45 (59)	-
Sex: F/M; *n.* (%)		17/14 (55/45)	22/23 (49/51)	0.647
Age: median (IQR), years		74 (64–84)	66 (52–79)	**0.036**
Diagnosis, *n.* (%)				
SE		17 (55)	13 (29)	**0.011**
CS		10 (32)	12 (27)	
SiS		4 (13)	20 (44)	
Pair-wise analysis	SE vs. CS			0.424
	SE vs. SiS			**0.003**
	CS vs. SiS			0.054
History of epilepsy		4 (13)	18 (40)	**0.011**
Acute symptomatic etiology		16 (52)	13 (29)	**0.045**
Time to MRI: median (IQR), hours		7 (7–46)	14 (5–32)	0.940

In bold: statistically significant differences. CS: cluster of seizures; PMA+: patients with peri-ictal MRI abnormalities; PMA−: patients without peri-ictal MRI abnormalities; SE: Status Epilepticus; SiS: single seizures.

**Table 4:** MRI findings at baseline according to the three groups.

	SE (*n* = 30)			CS (*n* = 22)			SiS (*n* = 24)		
	PMAs+	PMAs−	*p*	PMAs+	PMAs−	*p*	PMAs+	PMAs−	*p*
Patients, *n*. (%)	17 (57)	13 (43)		10 (45)	12 (55)		4 (17)	20 (83)	
Sex: F/M; *n*. (%)	9/8 (53/47)	7/6 (54/46)	0.713	7/9 (70/30)	9/3 (75/25)	0.793	1/3 (25/75)	7/13 (35/65)	0.699
Age: median (IQR), years	77 (66–84)	77 (65–84)	0.869	76 (70–87)	67 (49–81)	0.093	57 (40–70)	58 (44–70)	0.911
History of epilepsy, *n*. (%)	2 (12)	6 (46)	**0.049**	1 (10)	4 (33)	0.193	1 (25)	8 (40)	0.572
Acute symptomatic etiology, *n*. (%)	8 (47)	4 (31)	0.367	4 (40)	2 (17)	0.221	4 (100)	7 (35)	**0.031**
Status Epilepticus type, *n*. (%)									
Convulsive	7 (41)	4 (31)	0.662	-	-	-	-	-	-
Myoclonic	0	1 (8)		-	-		-	-	
Focal motor	7 (41)	6 (46)		-	-		-	-	
Non convulsive	3 (18)	2 (15)		-	-		-	-	
Seizure type, *n*. (%)									
Generalized	-	-	-	3 (30)	7 (58)	0.051	2 (50)	9 (50)	0.915
Focal	-	-		3 (30)	0		1 (25)	7 (33)	
Focal to bilateral	-	-		4 (40)	5 (42)		1 (25)	3 (11)	
Unknown	-	-		0	0		0	1 (6)	
Status Epilepticus etiology, *n*. (%)									
Acute	8 (47)	4 (31)	0.289	-	-	-	-	-	-
Remote	0	2 (15)		-	-		-	-	
Progressive	5 (29)	6 (46)		-	-		-	-	
In defined electroclinical syndromes	1 (6)	0		-	-		-	-	
Unknown	3 (18)	1 (8)		-	-		-	-	
Seizure etiology, *n*. (%)									
Structural	-	-	-	8 (80)	6 (50)	0.241	3 (83)	13 (68)	0.870
Genetic	-	-		0	0		0	1 (6)	
Metabolic	-	-		0	1 (8)		0	0	
Infectious	-	-		2 (20)	1 (8)		0	0	
Autoimmune	-	-		0	1 (8)		0	0	
Unknown	-	-		0	3 (25)		1 (17)	6 (33)	
SE duration: median (IQR), hours	24 (4–80)	36 (12–72)	0.594	-	-	-	-	-	-
SE duration > 24 h, *n* (%)	10 (59)	7 (54)	0.873	-	-	-	-	-	-
Time to MRI: median (IQR), hours	7 (7–40)	26 (18–40)	0.163	7 (7–85)	11 (3–32)	0.425	7 (7–36)	7 (4–27)	0.989

In bold: statistically significant difference. PMAs+: patients with peri-ictal MRI abnormalities; PMAs−: patients without peri-ictal MRI abnormalities.

## Data Availability

The datasets generated during and/or analyzed during the current study are available from the corresponding author on reasonable request.

## Supplementary Materials

The following supporting information can be downloaded at: https://www.mdpi.com/article/10.3390/jcm14082711/s1.

## Abbreviations

The following abbreviations are used in this manuscript:

CS	Clusters of seizures
CSE	Convulsive status epilepticus
ILAE	International League Against Epilepsy
NCSE	Non-convulsive status epilepticus
PMAs	Peri-ictal abnormalities
SE	Status epilepticus
SiS	Single seizures
