# Supplementary material for: Investigating Peri-Ictal MRI Abnormalities: A Prospective Neuroimaging Study on Status Epilepticus, Seizure Clusters, and Single Seizures
Source: J Clin Med. 2025 Apr 15;14(8):2711. doi: 10.3390/jcm14082711 (PMC12028061; doi:10.3390/jcm14082711)
Supplement: Supplementary file 1 [file jcm-14-02711-s001.zip › jcm-3524625-supplementary.pdf]

Table S1. Available literature data from prospective studies on PMA in SE

Study	Number of patients	MRI protocol	Time to MRI	SE duration	Incidence of PMA	Time to follow-up MRI	PMA resolution
Nair et al, 2009 [33]	99#	T1, T2, FLAIR	n.a.	76	69.1% (38/55)	Not performed.	-
Kalita et al, 2010 [32]	105*	T1, T2, FLAIR	n.a.	n.a.	62%	Not performed.	-.
Jabeen et al., 2017 [23]	23	DWI, ADC, FLAIR	<24 hours	12-72 hours	84.1% (15/29)	3 days–16 weeks (no established schedule)	100%<br>- complete: 80% (12/15)<br>partial 20% (3/15) -
Sarria-Estrada et al., 2022 [36]	60	T1, T2, FLAIR, DWI	<240 hours	26.4 hours (IQR 9.1-92.9)	51.7% (31/60)	3.2-17.4 months (mean: 9.7) after SE (no established schedule)	45% (15/33)
Bosque Varela et al, 2023 [4]	206	DWI, FLAIR, ASL	16 hours (median; IQR 0.25-373) from admission	n.a.	45% (93/206)	1 week, 4 weeks (scheduled MRI)	Total: 58/90 (64.4%).<br>-1 week: 39/66 (59%);<br>4 week: 19/24 (79%) -

Legend: ADC: apparent diffusion coefficient, ASL: arterial spin labeling, DWI: diffusion weighted imaging, FLAIR: fluid-attenuated inversion recovery, n.a.: not available, PMA: peri-ictal MRI abnormalities , SE: status epilepticus. *MRI performed in 61 patients (only cranial CT in the remaining 44); #MRI performed in 55 patients (only cranial CT in the remaining 44). Refer to the main text for references.

Table S2. Relevant literature data on PMA in CS and SiS

Study	Type of study	Number of patients	MRI protocol	Time to MRI	CS/SiS duration	Incidence of PMA	Time to follow-up MRI	PMA resolution
Hufnagel et al, 2003 [20]	prospective	9 SiS	DWI, ADC	2-210 minutes	20-420 seconds	66.7% (6/9)	Not performed	-
Raghavendra et al, 2007 [31]	retrospective	1700 CS, SiS	T1, T2, FLAIR	n.a.	n.a.	0.007%	10 days - 60 months (no established schedule)	100%
Cianfoni et al., 2013 [21]	retrospective	26 SE, CS, SiS	T2, FLAIR, DWI, ADC, Gadolinium-enhancement	<7 days	n.a.	n.a.	15 -150 days -average: 62 days – (no established schedule)	complete: 15/26 partial: 11/26
Xiang et al., 2014 [22]	retrospective	~ 300 CS and SiS	DWI, ADC, FLAIR	<14 days	n.a.	14 (5 CS, 9 SiS)	<45 days (no established schedule)	85.7% - complete 71.4% (10/14), partial 7.1% (1/14) -
Jabeen et al., 2017 [23]	prospective	6 CS	DWI, ADC, FLAIR	<24 hours	26-96 hours	83.3% (5/6)	1-5 weeks (no established schedule)	100% - complete: 80% (4/5), partial 20% (1/5) -
Hübers et al, 2018 [24]	retrospective	454 SE, CS, Sis (416 SiS)	DWI, ADC	<24 hours	n.a.	CS: n.a. SiS: 3% (11/454)	Not performed	-

Legend: ADC: apparent diffusion coefficient, ASL: arterial spin labeling, CS: cluster of seizure, DWI: diffusion weighted imaging, FLAIR: fluid-attenuated inversion recovery, n.a.: not available, PMA: peri-ictal MRI abnormalities, SE: status epilepticus, SiS: single seizure. Refer to the main text for references.

Supplementary Figures

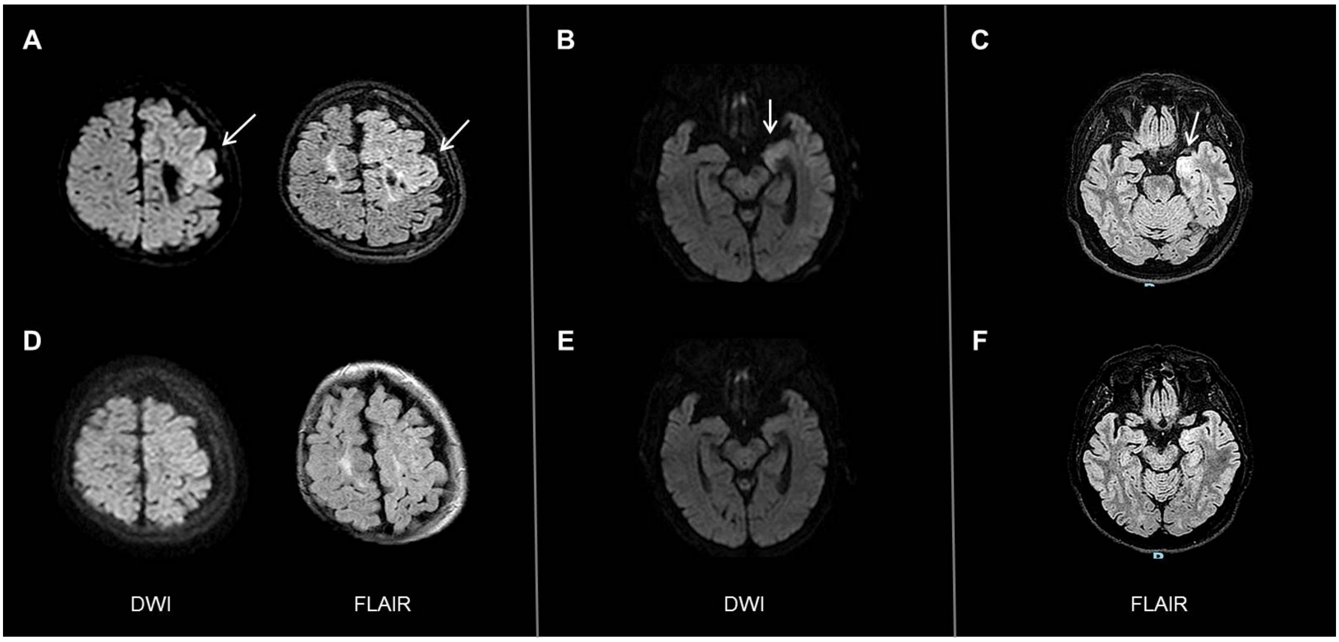

Figure S1. Examples of PMA in three patients with Status Epilepticus (SE), cluster of seizures (CS) and single seizure (Sis) patient at baseline and at follow-up.

The figure shows MRI imaging of three patients showing PMA (white arrows) after ictal event and subsequent complete resolution at the follow-up MRI.

The first patient was a 18-year-old male with profound mental disability (unknown cause), bedridden with severe spastic tetraparesis and fed by means of percutaneous gastrostomy since age 16; he had his first tonic-clonic seizure at age 18 months, followed by monthly seizures characterized by right head deviation, involuntary facial movements, and forced smiling, treated with phenobarbital. The patient was hospitalized for focal motor status epilepticus (seizures with the same semiology). MRI performed 7 hours after SE (A) showed (apart from hypoplasia of the corpus callosum with dilation of the lateral ventricles and enlargement of the left Sylvian fissure: not shown) FLAIR hyperintensity and signs of restricted diffusion (DWI) in some cortical gyri of the left frontal convexity, with signs of cortical swelling. MRI performed 2 weeks later (D) showed improvement of the abnormalities both in DWI and FLAIR sequences.

The second patient was a 89-year-old man with hypertension, diabetes and atrial fibrillation. During a few hours, he suffered some episodes of altered speech followed by full recovery and another seizure characterized by right head and gaze deviation, shaking of the head and right limbs, followed by global aphasia and right-sided paresis that lasted 24 hours. MRI just after the tonic-clonic seizure (B) showed signal alteration, with signs of restricted diffusion coefficient (DWI) more evident in the left para-hippocampal and mesial temporal regions; further signal alteration with similar characteristics seems to be present also in the left thalamic region (not shown). MRI performed five days later (E) showed almost complete resolution (DWI).

The third patient was a 60-year-old man with hypertension diabetes mellitus. He came to observation for a history of seizures characterized by a sensation of heat radiating from the nape to the face, accompanied by mild skin flush. 1. A MRI performed 2 days after a seizure (C) showed signal alteration with subtle hyperintensity on T2-dependent images (FLAIR), with an oedematous-like appearance, localized in the left para-hippocampal temporal region. Lacosamide was administered with good seizure control. A MRI performed 2 months later (F) showed improvement of the signal abnormality.

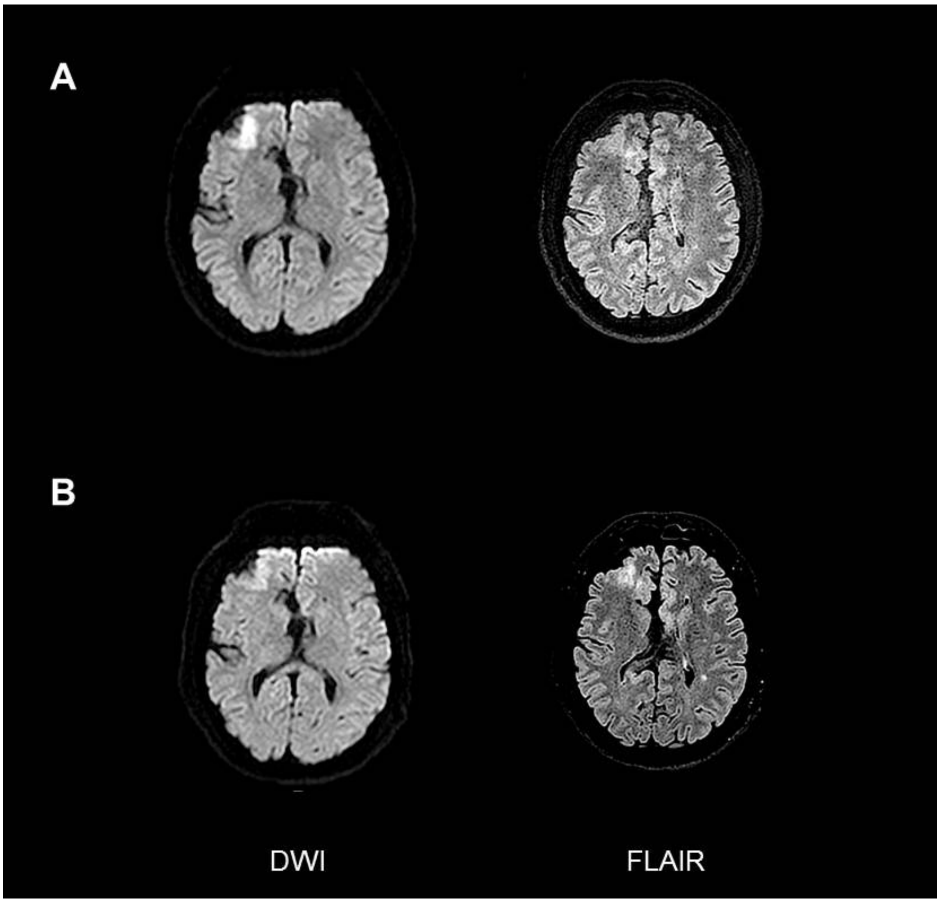

Figure S2. An unusual PMA image.

The figure shows MRI imaging of a 48-year-old male patient with a known complex malformation (partial agenesis of the corpus callosum, polymicrogyria of both frontal cortices and right cingulate gyrus) and drug resistant epilepsy. He came to our observation after a focal to bilateral tonic-clonic seizure. MRI performed 7 hours after the event (A), showed restriction in right frontal cortex (DWI) with concomitant oedema and polymicrogyria in the same site (FLAIR). MRI performed 7 days later (B), showed decreased restriction (DWI) as well as reduced oedema (proving to be PMA), but persistence of abnormal gyration (FLAIR).
